# Supplementary material for: Automated Risk Prediction of Post-Stroke Adverse Mental Outcomes Using Deep Learning Methods and Sequential Data
Source: Bioengineering (Basel). 2025 May 14;12(5):517. doi: 10.3390/bioengineering12050517 (PMC12109392; doi:10.3390/bioengineering12050517)
Supplement: Supplementary file 1 [file bioengineering-12-00517-s001.zip › bioengineering-3539681-supplementary.pdf]

Supplementary Materials

Table S1: List of features collected and used for modelling

No.	Categories	Features	Data Type	Description	Remarks
1	Demographics	Gender	Binary	Patient’s gender	• Features are collected during the admission date
2		Race	Categorical	Patient’s race	
3		Age at admission	Continuous	Patient’s age at point of admission	
4		Marital Status	Categorical	Patient’s marital status at point of admission	
5		Living Arrangement	Categorical	Who is staying together with the patient	
6		History of Depression	Binary	Indicator if patient has a history of depression	
7		Other Psy. History	Binary	Indicator if patient has other psychiatric history	
8		Family Psy. History	Binary	Indicator if patient’s family has other psychiatric history	
9		Education Level	Categorical	Patient’s education level	
10		Housing Type	Categorical	Patient’s type of housing	
11		Occupation	Categorical	Patient’s occupation	
12		Alcohol Use	Categorical	Patient’s alcohol consumption habits	
13		Drug Use	Categorical	Patient’s drug consumption habits	
14		Smoking	Categorical	Patient’s smoking habits	
15	Stroke Information	Stroke Incidence	Categorical	Indicator if the admission is the incidence occurrence of stroke	• Features are collected during the admission date
16		Stroke Laterality	Categorical	Side of the brain affected by stroke	
17		Nature of Stroke	Categorical	Ischemic, Haemorrhagic, etc.	
18		Structure of Stroke	Categorical	Thalamus, Basal Ganglia, etc.	
19		Stroke Circulation	Categorical	Posterior, Anterior, etc.	

20		Type of Stroke	Categorical	Arteriovenous malformation (AVM), small vessel disease (SVD), and large vessel disease (LVD), etc.	
21	Medication History	Antidepressants	Binary	Indicator if patient has consumed specific drugs	• Searched for patient's lab results from 365 days before admission date to 3 days after admission date.
22		Anti-psychotics	Binary		
23		Benzodiazepines	Binary		
24		Dementia	Binary		
25		Mood Stabilizers	Binary		
26		Others	Binary		
27	Surgical History	AVMAneu	Binary	Indicator if patient has gone through specific type of surgery	• Searched for patient's lab results from 3 years before admission date to 3 days after admission date.
28		Craniectomy	Binary		
29		Craniotomy	Binary		
30		Cranioplasty	Binary		
31		Others	Binary		
32	Test scores	AMT	Ordinal	Abbreviated Mental Test score	• Features are collected during the admission date
33		EQ5D-VAS	Ordinal	Patient's EQ-5D Visual Analog Scale (VAS) score, measuring his health-related quality of life	
34		FIM – Cognitive Score	Ordinal	Patient's Functional Independence Measure (FIM) Score, Cognitive component	
35		FIM – Motor Score	Ordinal	Patient's Functional Independence Measure (FIM) Score, Motor component	
36	Laboratory Results	Basophils	Continuous	Patient's lab results taken closest to admission	• Searched for patient's lab results from 365 days before admission date to 3 days after admission date.<br>• Selected the results closest to the admission date
37		Creatinine	Continuous		
38		Eosinophils	Continuous		
39		Haemoglobin	Continuous		
40		Lymphocytes	Continuous		
41		Potassium	Continuous		
42		Red blood cell count	Continuous		
43		Red blood cell width	Continuous		

44		Sodium	Continuous		
45		White blood cell count	Continuous		
46	Psy. Intervention History	Psy. Intervention Services done	Binary	Indicator if patient has been through any psychiatric intervention	• Searched for patient's medication records from 3 years before admission date to 3 days after admission date
47	Daily Laboratory Results *(7-day Time Series)* *"Referred to as sequential data in this study"*	Basophils	Continuous	Patient's lab results taken at the start of the day	• Searched for the patient's laboratory results from admission date to 7 days after admission date.<br>• Results are extracted daily
48		Basophils Percentage	Continuous		
49		Creatinine	Continuous		
50		Eosinophils	Continuous		
51		Eosinophils Percentage	Continuous		
52		Haematocrit	Continuous		
53		Haemoglobin	Continuous		
54		Lymphocytes	Continuous		
55		Lymphocytes Percentage	Continuous		
56		Monocyte	Continuous		
57		Monocyte Percentage	Continuous		
58		Neutrophils	Continuous		
59		Neutrophils Percentage	Continuous		
60		Platelets	Continuous		
61		Potassium	Continuous		
62		Red blood cell count	Continuous		
63		Red blood cell width	Continuous		
64		Sodium	Continuous		
65		White blood cell count	Continuous		
